# Utility of a Novel Mobile Lip‐Reading Application for Patients After Total Laryngectomy

**DOI:** 10.1002/ohn.70098

**Published:** 2026-01-04

**Authors:** Carly A. Fassler, Shreyas G. Krishnapura, Daniel Sharbel, Eben Rosenthal, James Netterville, Kyle Mannion, Alexander Langerman, Robert Sinard, Sarah Rohde, Michael C. Topf

**Affiliations:** ^1^ Vanderbilt University School of Medicine Nashville Tennessee USA; ^2^ Department of Otolaryngology–Head & Neck Surgery Augusta University Medical Center Augusta Georgia USA; ^3^ Department of Otolaryngology–Head and Neck Surgery Vanderbilt University Medical Center Nashville Tennessee USA; ^4^ School of Engineering Vanderbilt University Nashville Tennessee USA

**Keywords:** head and neck cancer care, novel technology, patient quality of life, post‐operative communication, Total laryngectomy

## Abstract

Total laryngectomy (TL) patients have limited options for speech rehabilitation in the immediate postoperative period, resulting in communication difficulties and patient frustration. This study investigates a novel mobile application (app), Speech Recognition Application for the Voice Impaired (SRAVI), to improve postoperative communication for TL patients. Participants received a tutorial on the technology prior to use and had access to the app throughout their hospital admission. A total of 16 patients were included. Of 652 valid transactions within the app, accurate phrase return on the first attempt was 43% with a total accuracy of 67%. Patients used SRAVI primarily to communicate with their care team (66.7%) and with family members/social support (80%). Patients reported using SRAVI, on average, 43.6% of the time to communicate during their hospitalization the majority (69.7%) preferred SRAVI to written communication. In TL patients, use of speech recognition technology is feasible in the immediate postoperative period.

Total laryngectomy (TL) is performed upfront for locally advanced T4 laryngeal cancer, as salvage surgery for those with recurrent disease, and for dysfunctional larynx following definitive chemoradiation therapy (CRT).^1^ Patients often struggle with the morbidity that occurs as a result of this procedure, including communication difficulties and psychological distress.^2^, ^3^, ^4^ The loss of natural voice is associated with poorer quality of life and psychological discomfort following TL.^3^, ^4^

Several methods of voice rehabilitation are available following TL, including esophageal speech (ES), tracheoesophageal speech (TES), and electrolarynx speech (ELS)^5^, ^6^ However, not all patients have access to these methods^7^ or have positive outcomes using voice prostheses.^8^ ES and TES rehabilitation typically cannot take place until some healing has occurred, therefore TL patients are left without audible speech for a period of time postoperatively. ELS requires a substantial learning curve and demands a certain level of neurocognitive capability in order to be used effectively.^9^ Given these limitations, novel mechanisms for speech in the immediate postoperative laryngectomized patient are needed. Prior studies have investigated various methods for silent speech recognition including implantable magnets,^10^, ^11^ surface EMG,^12^, ^13^ and intraoral recording devices.^14^ However, many of these studies were performed in a controlled research environment or only on healthy controls.

This article explores the use of an intuitive speech recognition solution, Speech Recognition Application for the Voice Impaired (SRAVI), a mobile application (app) that recognizes short lip movements using artificial intelligence (AI) and converts them to audible speech.

## Methods

This prospective study was approved by Vanderbilt University Medical Center Institutional Review Board (IRB #220085). Patients undergoing TL between October 2022 and March 2024 for laryngeal malignancy or dysfunctional larynx were included.

SRAVI is a mobile app that converts lip movements to speech. The app was developed by Liopa (Belfast, Ireland) in collaboration with staff at Lancashire Teaching Hospitals Trust (Lancashire, England). SRAVI allows a patient to record a video of a short lip movement and recognizes the phrase using artificial intelligence. The video is sent from the app to the server and a list of three potential recognition candidate phrases are returned and displayed. When the user selects a phrase, text‐to‐speech technology is used to audibly output the phrase. Prior to implementation of this study, SRAVI was primarily utilized in intensive care unit patients, including in tracheostomy, trauma, and stroke patients. Results of the initial feasibility study demonstrated total phrase‐recognition accuracy of 74% to 86%.^15^ At the time of the current study, the SRAVI app was free to download for users testing it in the hospital setting.

No patient identifying information was collected by Liopa via the SRAVI application other than video data containing patients' facial features. All video data stored by SRAVI was encrypted both in transit and when stored on the SRAVI servers. Patient data was anonymized using a generic username and no identifying patient details were stored including their name.

Patients were enrolled on postoperative Day 1. The app was loaded onto an iPad tablet (Apple). Participants received a tutorial on SRAVI and the tablet prior to use and had access to the app for the duration of their admission. Research personnel spent 15 to 30 minutes to provide a tutorial on the use of SRAVI on postoperative Day 1 and returned once per day during the duration of the patient's hospitalization, on average spending 5 to 10 minutes per day over the course of 7 to 10 days.

At discharge, participants completed a modified version of the Self‐Evaluation of Communication Experiences After Laryngeal Cancer (SECEL), a validated self‐evaluation measure of communication impairment following TL,^16^ the 10‐item Ease of Communication Scale,^17^, ^18^ and a custom survey regarding their experience using SRAVI. The SECEL survey used in the current study was modified from the original SECEL to remove questions not applicable to patients who have not yet been discharged from the hospital. The SECEL survey has been validated in addressing communication dysfunction in laryngectomized patients.^19^ The SRAVI feedback survey was developed by our team as a way to capture feedback on the technology from patients in this preliminary study. Respective survey instruments are found in Supplemental Figures S1‐3, available online. All data were collected and stored using a HIPPA‐compliant data management system (REDCap).

## Results

### Patient Demographics

Of the 16 patients included in the study, the majority were male (73%) and White (93%). Mean age of the cohort was 66.1 ± 5.8. Patients underwent TL as treatment for active malignancy (80%) or dysfunctional larynx due to prior history of CRT to the head and neck (20%). Most patients (86.7%) had a preoperative visit including TL counseling while the remainder (13.3%) underwent TL during an inpatient admission.

### SRAVI Accuracy Data

Of 652 valid transactions within the app, the correct phrase was generated on the first attempt 43% of the time. The correct phrase was generated within the first three attempts 67% of the time. Table 1 displays the accuracy data for the first three attempts at generating a phrase across all patients.

**Table 1:** Accuracy Data: Return for First Three Attempts at Generating a Phrase, Rank‐1 Accuracy, and Total Accuracy Across all Patients

Patient	Return 1 (R1)	Return 2 (R2)	Return 3 (R3)	Total valid transactions	Rank‐1 accuracy (R1 + R2 + R3)	Total accuracy
1	5	3	1	11	45.45%	81.82%
2	5	2	0	7	71.43%	100.00%
3	13	8	6	51	25.49%	52.94%
4	16	8	5	37	43.24%	78.38%
5	5	6	2	31	16.13%	41.94%
6	24	13	13	83	28.92%	60.24%
7	43	9	3	73	58.90%	75.34%
8	7	1	1	25	28.00%	36.00%
9	4	1	1	6	66.67%	100.00%
10	6	2	1	15	40.00%	60.00%
11	3	0	0	4	75.00%	75.00%
12	20	7	6	57	35.09%	57.89%
13	3	2	1	8	37.50%	75.00%
14	6	2	2	32	18.75%	31.25%
15	32	11	7	51	62.75%	98.04%
16	87	25	6	161	54.04%	73.29%
Total	**279**	**100**	**55**	**652**	**43%**	**67%**

R1, R2, R3 refers to whether the correct phrase was generated on video attempt 1, 2, or 3, respectively. Rank‐1 Accuracy refers to what percent of the time the phrase was correct on the first try out of the total number of transactions. Total Accuracy refers to what percent of the time the phrase was correct on the first three tries (R1 + R2 + R3) out of the total number of transactions.

A Pearson correlation test was conducted to examine the association between age and rank‐1 accuracy. Analysis revealed a negative correlation in accuracy with increasing age that was not significant (*r*(14) = −0.37, *P* = .16).

### Modified SECEL/Ease of Communication Scale Surveys

**Figure 1:**
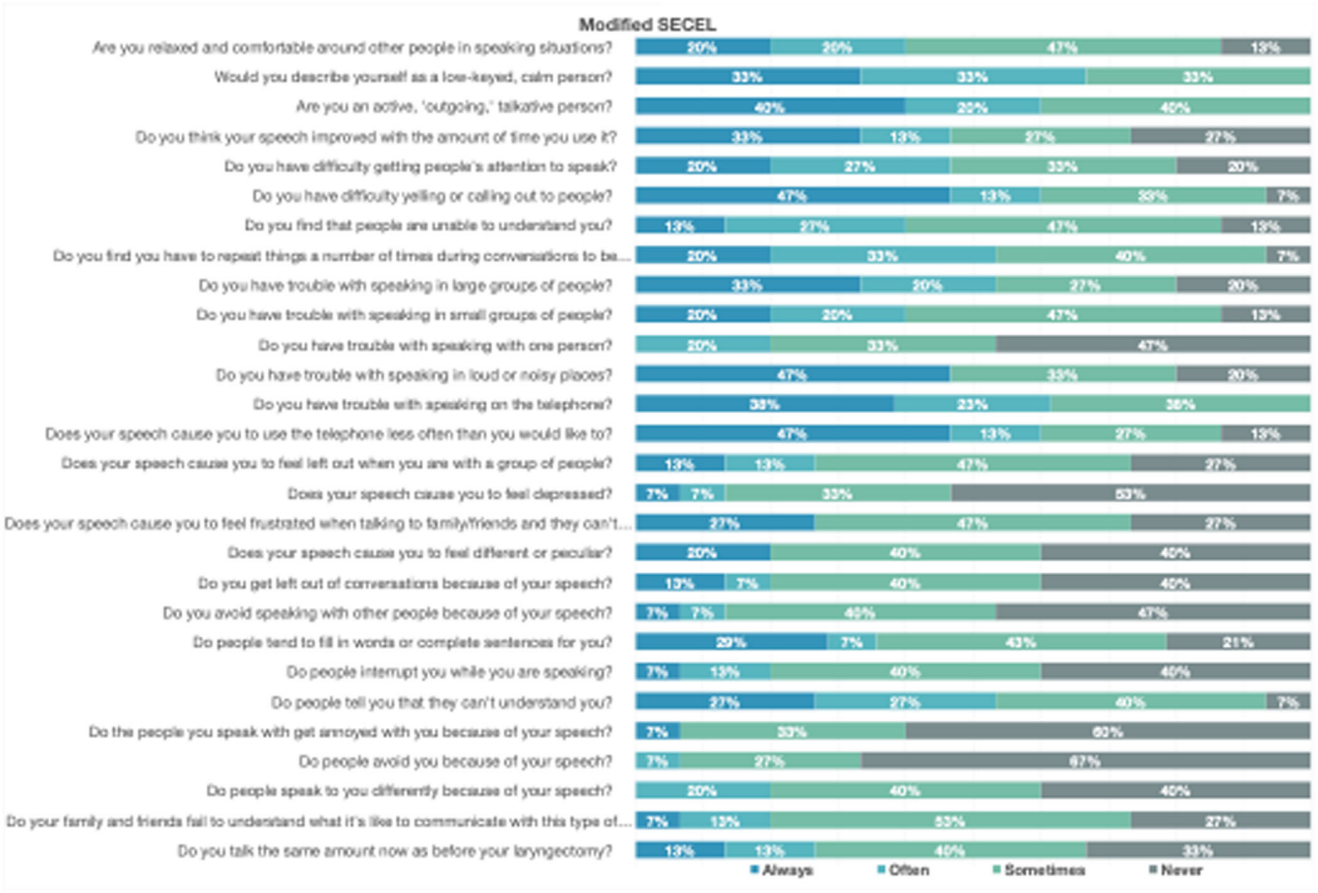
Modified Self‐Evaluation of Communication Experiences After Laryngectomy (SECEL): patient responses on the day of hospital discharge following total laryngectomy.

**Figure 2:**
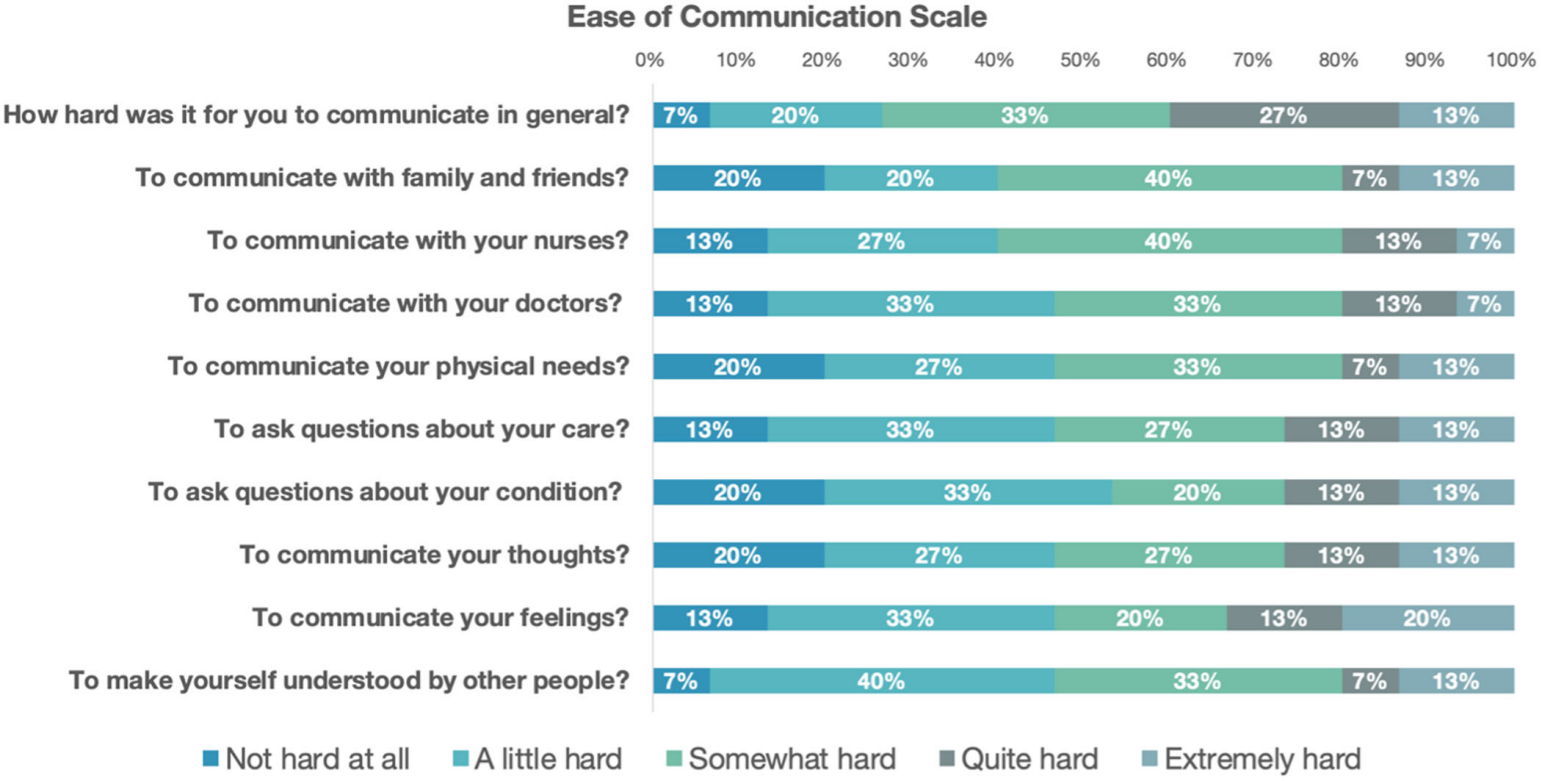
Ease of Communication Scale: Patient responses on the day of hospital discharge following total laryngectomy.

All 16 patients completed the modified SECEL and Ease of Communication Scale questionnaires. In general, patients reported the most difficulty with yelling or calling out to others (60%), speaking in loud environments (80%), and speaking on the telephone (61%). Patients reported feeling frustrated when communicating with social support (81%) and that their family or friends don't understand what it's like to communicate with a speech impairment (73%). Full cohort responses for both questionnaires are shown in Figures 1 and 2.

### Custom Survey

All 16 patients completed the SRAVI experience survey. Patients found SRAVI helpful in communicating during their hospital stay (mean response 7.87 on a scale of 0‐10, 10 being most helpful) and easy to use (8.4). Patients primarily used SRAVI to communicate with their care team (66.7%) and with family/social support (80%). Patients reported using SRAVI, on average, 43.6% of the time to communicate during their hospitalization, and 69.7% of participants preferred SRAVI to written communication (Supplemental Table S1, available online).

## Conclusion

SRAVI, a visually based speech recognition app, may be useful to improve communication for patients in the immediate postoperative period following TL. Patients were able to successfully use SRAVI to communicate with their care team and family members, when they would otherwise need to rely on alternate communication methods such as writing or gesturing. Patients had a generally positive experience with this technology and provided valuable feedback on ways similar technology could be improved in the future. This study was conducted in the inpatient setting with minimal coaching by research personnel, therefore, this technology may be a viable option for patients to use independently at home.

While writing may provide 100% communication accuracy for most patients, it has several limitations. Unfortunately, not all patients are literate and able to communicate effectively through written word. Additionally, writing out thoughts, feelings, and questions can be tedious and time‐consuming for patients, especially in the setting of decreased strength following major head and neck surgery or in patients with impaired motor function. Finally, we feel that the psychosocial benefit of communicating with spoken voice versus using a writing pad may be beneficial in this population. Future studies comparing traditional methods of communication in this patient population versus lip‐reading technology are warranted to further characterize the utility of this method.

We recognize the limitations of our study. SRAVI was trialed in the inpatient setting due to restrictions on ability to provide the technology to patients ahead of time as well restrictions on study personnel. Enrolling patients preoperatively and allowing them to trial SRAVI in the preoperative setting would allow them to practice with the technology and potentially lead to better outcomes. Additionally, in the current study, SRAVI used a fixed phrase library. However, with current advancements in AI technology, free‐text recognition will be a possibility for this technology in the future. The study cohort demographics align with the population of patients seen at our institution, which are majority White and English‐speaking. This is a limitation of our study population, and future studies in a more diverse population are warranted. Finally, we did not obtain feedback from nursing staff; however, it would be beneficial to include feedback from nursing and other members of the multidisciplinary care team in future studies to further understand the impact of this technology on daily patient care.

## Author Contributions

**Carly Fassler**: conduct, analysis, final manuscript writing; **Shreyas Krishnapura**: design, conduct, analysis, final manuscript writing; **Daniel Sharbel**: conduct, analysis, final manuscript writing; **Eben Rosenthal**: design, conduct, analysis, final manuscript review; **James Netterville**: design, conduct, analysis, final manuscript review; **Kyle Mannion**: design, conduct, analysis, final manuscript review; **Alexander Langerman**: design, conduct, analysis, final manuscript review; **Robert Sinard**: design, conduct, analysis, final manuscript review; **Sarah Rohde**: design, conduct, analysis, final manuscript review; **Michael C. Topf**: design, conduct, analysis, final manuscript writing and review.

## Disclosures

### Competing interests

None.

### Funding source

This work was supported by a Vanderbilt Clinical Oncology Research Career Development Program (K12 NCI 2K12CA090625‐22A1).

## Supporting information


**Supplemental Table1:** Qualitative feedback collected from participants following use of the SRAVI app. **Supplemental Figure1**: Modified Self‐Evaluation of Communication Experiences (SECEL) after Laryngectomy Survey Instrument. **Supplemental Figure2**: Ease of Communication Scale Survey Instrument. **Supplemental Figure 3**: Speech Recognition Application for the Voice Impaired (SRAVI) Patient Experience Survey Instrument.
