# Supplementary material for: Utility of a Novel Mobile Lip‐Reading Application for Patients After Total Laryngectomy
Source: Otolaryngol Head Neck Surg. 2026 Jan 4;174(2):582–6. doi: 10.1002/ohn.70098 (PMC12860178; doi:10.1002/ohn.70098)
Supplement: Supplementary file 1 — Supplemental Table1: Qualitative feedback collected from participants following use of the SRAVI app. Supplemental Figure1: Modified Self‐Evaluation of Communication Experiences (SECEL) after Laryngectomy Survey Instrument. Supplemental Figure2: Ease of Communication Scale Survey Instrument. Supplemental Figure 3: Speech Recognition Application for the Voice Impaired (SRAVI) Patient Experience Survey Instrument. [file OHN-174-582-s001.docx]

**SUPPLEMENT:**

| ***Participant Qualitative Feedback*** |
| --- |
| *“It’s amazing! We enjoyed using the app”* |
| *“Get it on the market.”* |
| *“It is fantastic! I really enjoyed using the app”* |
| *“A very good thing for people like mother”* |
| *“It was a little frustrating at the beginning when he would try to use the app and it wouldn't understand his lip movements It is very easy to use, but he wishes that the accuracy level was higher so that he wouldn't become frustrated”* |
| *“Application does not understand movement of my lips and face very well, I have to over-enunciate for it to understand. Perhaps my facial hair gets in the way of it reading my lips. if I don't enunciate clearly it doesn't understand. I have to enunciate more than usual”* |
| *“If the app could recognize my lip movements through the swelling that would have helped”* |
| *“I think the app is a very good idea, but it did not work well for me”* |
| *“I think it is great, just add more phrases to it”* |
| *“A little challenging to use at night or in lower light. Sometimes couldn't catch everything i wanted to say, but it is a very good idea and easy to use”* |

**Supplemental Table 1:** Qualitative feedback collected from participants following use of the SRAVI app.

**Supplemental Figure 1**: Modified Self-Evaluation of Communication Experiences (SECEL) after Laryngectomy Survey Instrument.

**Modified SECEL Survey**


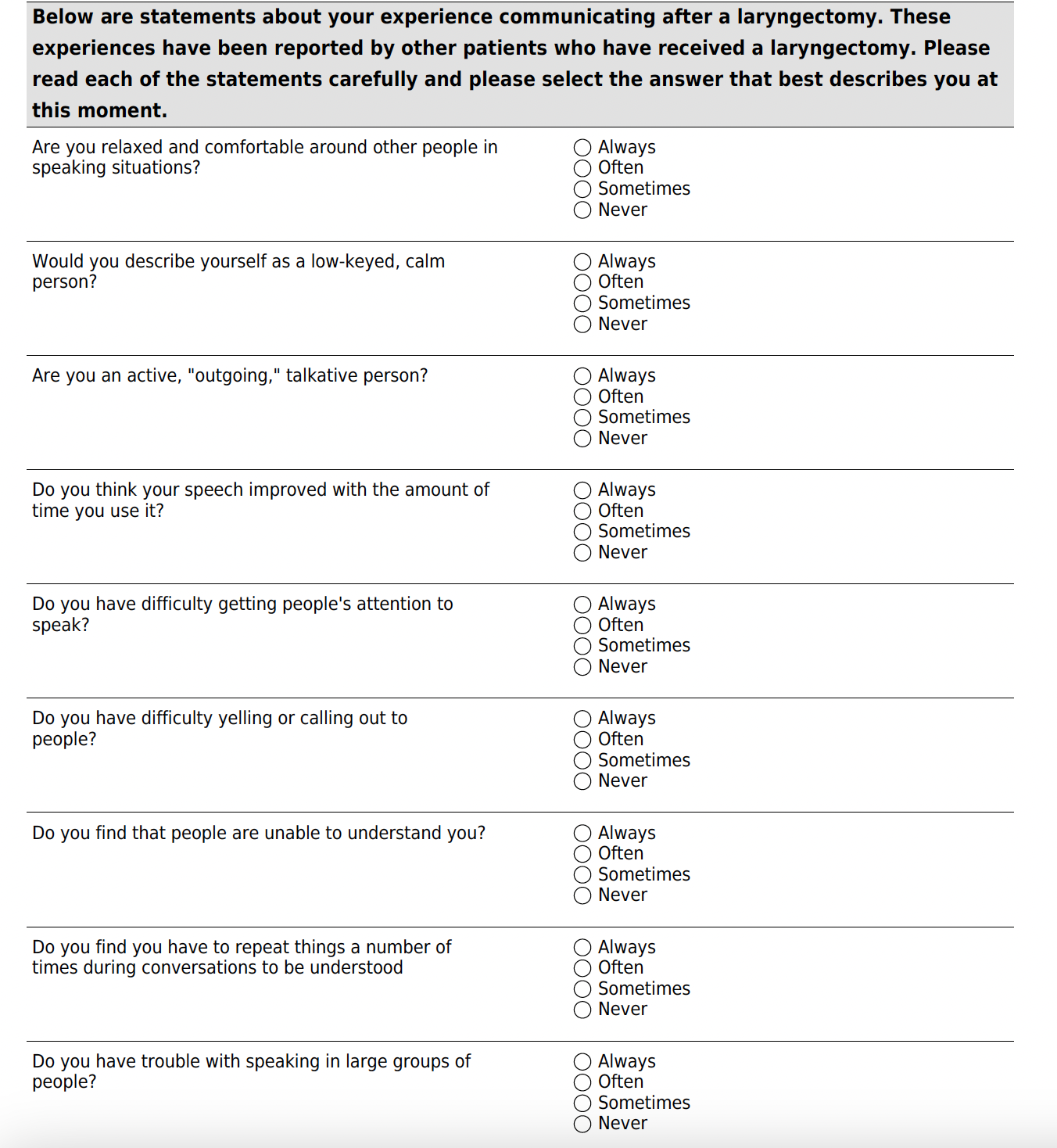


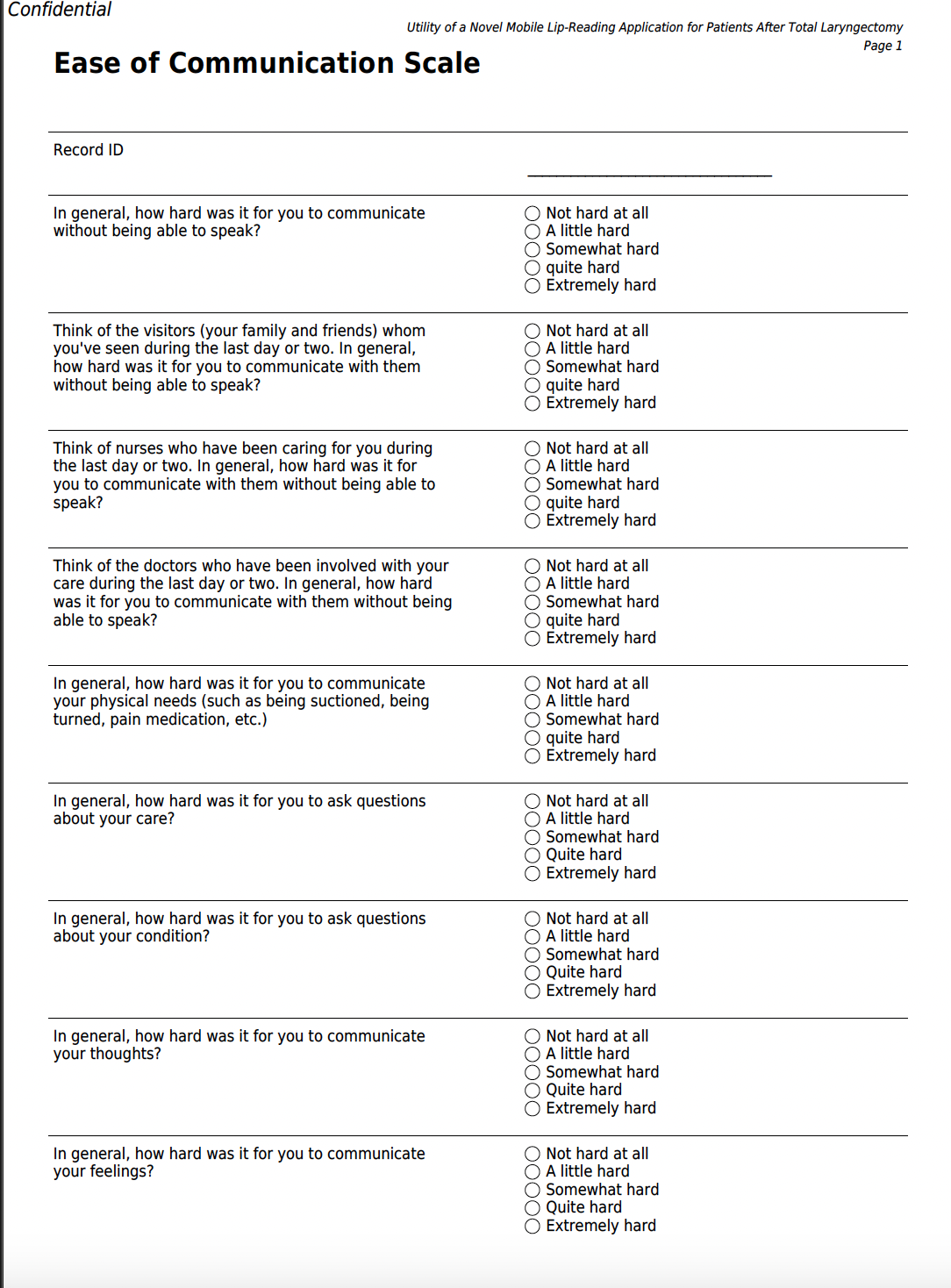


**Supplemental Figure 2**: Ease of Communication Scale Survey Instrument.

**
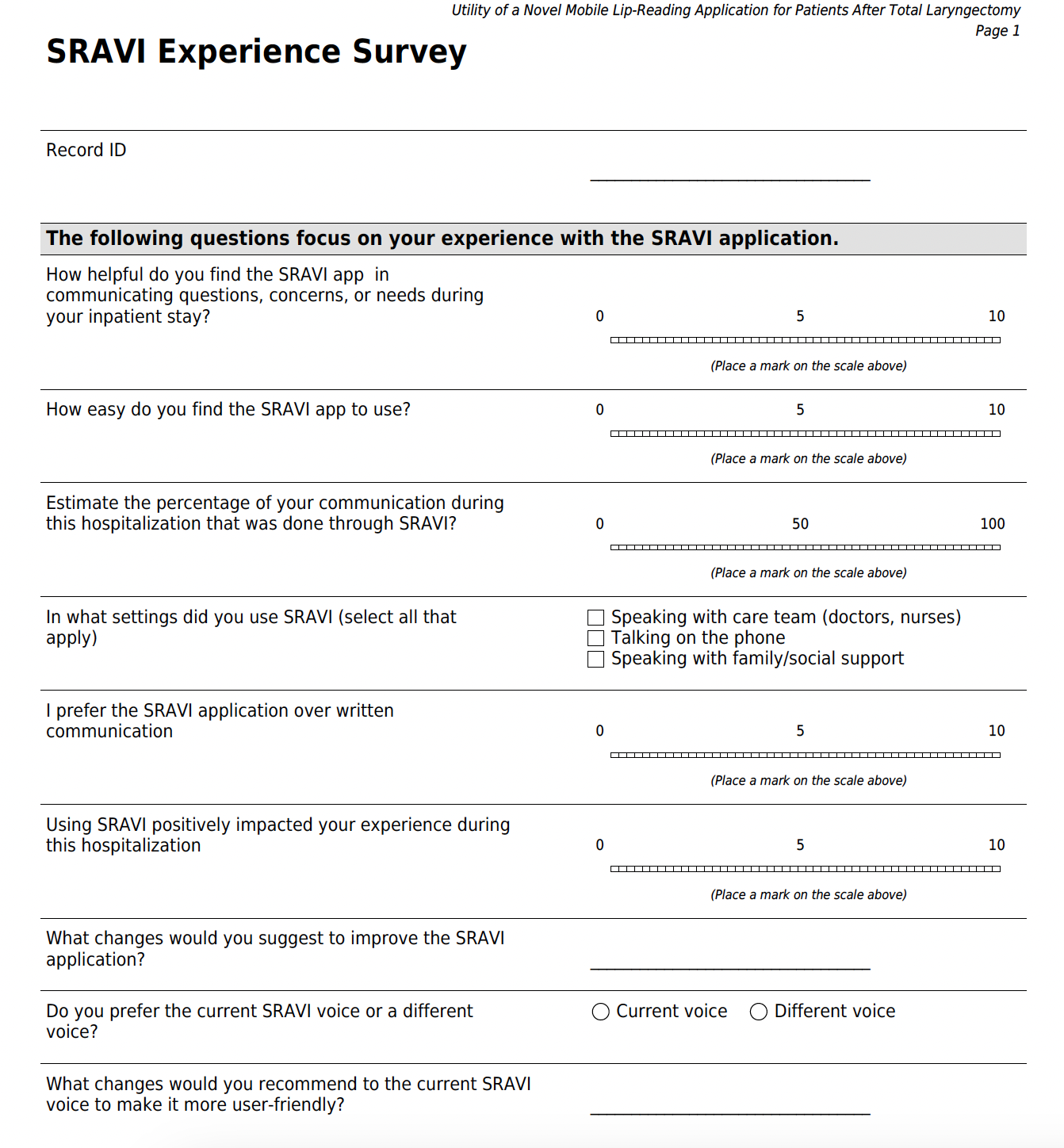
Supplemental Figure 3**: Speech Recognition Application for the Voice Impaired (SRAVI) Patient Experience Survey Instrument.
